# Personalisation and social care assessment – the Care Act 2014

**DOI:** 10.1192/pb.bp.116.053660

**Published:** 2017-06

**Authors:** Deb Barnes, Billy Boland, Kathryn Linhart, Katherine Wilson

**Affiliations:** 1 Hertfordshire Partnership NHS Foundation Trust

## Abstract

The Care Act 2014 represents a significant change in legislation in England. For the first time it brings together various aspects of adult social care into a single statute succeeding earlier acts and policy. Given its importance to the lives of service users and carers, clinicians need to have a clear understanding of its implications. We provide an overview of why it was developed, its underlying principles and international comparisons, as well as implications for assessments, interventions and outcomes. The impact on the lives of patients and carers is discussed, as well as dilemmas and challenges the Act presents. While it addresses other important aspects of social care, including safeguarding, Mental Health Act section 117 aftercare and duty of candour, we focus on personalisation because of the opportunities it provides to enhance management plans for people experiencing mental health problems.

## Why was the Care Act 2014 developed?

The Care Act 2014 represents the latest evolution in current attitudes to care. It was asserted by Norman Lamb MP, Care and Support Minister in the UK coalition government, as ‘the most significant reform of care and support in more than 60 years’.^1^ Think Local Act Personal (TLAP, a partnership of more than 50 organisations, including the National Health Service (NHS), ‘committed to transforming health and care through personalisation and community-based support’(www.thinklocalactpersonal.org.uk/About-us/)) sees it as representing a significant change in legislation, of importance to service users and carers in England and Wales because ‘for the first time it puts them in control of their care and support. It also makes clear what kind of care they should expect’ (www.thinklocalactpersonal.org.uk/Browse/careact2014/).

Social care law can be seen to have begun with the National Assistance Act in 1948 that contained provisions for the basis of the modern welfare state. Direct payments as a method of personalisation were introduced in 1996 by the Community Care Act. More recent updates, such as the Carers and Disabled Children Act 2000 and the Health and Social Care Act 2001, broadened and refreshed these approaches. Separately, safeguarding of adults from abuse was dealt with by the Department of Health in *No Secrets*.^2^ The Care Act 2014 succeeds earlier acts with a single statute. The golden thread running through the Act is the promotion of individual well-being (Box 1):

‘The general duty of a local authority, in exercising a function under this Part in the case of an individual, is to promote that individual's well-being.’ (Care Act 2014 section 1(1))

‘As a service user who has in the past been a carer to a diverse group of individuals, I feel that had a lot of these changes been made in the past, many people's lives would have transformed sooner rather than later. That being said, we are now moving forward to enable individual lives to be more fulfilled.’ (Deb Barnes)

**Box 1** Scope: purpose of the Care Act (adapted from the Care Act 2014)

- To reform the law relating to care and support for adults
- To reform the law relating to support for carers
- To make provision about safeguarding adults from abuse or neglect
- To make provision about care standards
- To establish and make provision about Health Education England
- To establish and make provision about the Health Research Authority
- To make provision about integrating care and support with health services

**Box 2** Care Act specified outcomes

The specified outcomes as defined in the Act are:

- managing and maintaining nutrition
- maintaining personal hygiene
- managing toilet needs
- being appropriately clothed
- being able to make use of the adult's home safely
- maintaining a habitable home environment
- developing and maintaining family or other personal relationships
- accessing and engaging in work, training, education or volunteering
- making use of necessary facilities or services in the local community including public transport and recreational facilities or services, and
- carrying out any caring responsibilities the adult has for a child.

## What are the international comparisons?

In establishing the Care Act, a cap on spending for long-term social care was originally proposed to be introduced in April 2016. However, this has now been postponed until at least 2020. In 2014, The King's Fund reviewed international comparisons for health and social care provision.^3^ It highlights that The Netherlands introduced a ‘universal’ (i.e. available to all) system of insurance for long-term care in 1968. In the 1990s it introduced caps in response to rising costs, but this led to long waiting lists and the caps were abolished. Latterly, they have raised the threshold to access social care and outlined aspects of care that are expected to be delivered by families. Sweden established the right to tax-funded legislation in 1982/1983, whereas countries including Germany, France, Japan and South Korea all have mandatory long-term care insurance schemes.

## Assessment under the Care Act

Assessment of needs, both for service users and their carers, is a core aspect of the Care Act. The process is divided into three stages: identifying needs, assessing eligibility and care planning.^4^ Each of these should be viewed not only as a gateway to support but as an intervention in itself.

### Stage 1: identifying needs

The Act places a statutory duty on local authorities to provide assessments for any adult, including carers, appearing to have a need for care or support, regardless of the local authority's view of the level of that need or the individual's financial resources (section 9–10). It is important to note that in some areas this responsibility may be delegated by the local authority to partner organisations, such as NHS foundation trusts, and that assessment may be carried out by a range of professionals, including healthcare professionals. This assessment should address the person's needs in relation to the specified outcomes as defined in the Care Act (Box 2). It aims to identify the impact of these needs, the person's desired outcomes, and whether the provision of care and support services will be effective in contributing to the achievement of these outcomes (section 9(4), 10(5)). For clarity, we have used the term ‘specified outcome’ as a technical definition to refer to those outcomes specified in the Care Act (Box 2) and ‘personal outcome’ to describe all possible outcomes individuals may see as important.

It is crucial that the individual, their carer and any other person the individual requests be fully involved in the assessment process. Consideration should also be given to the most appropriate kind of assessment. Options for supported self-assessment, telephone assessment, joint assessment with other agencies or a combined assessment of the needs of, for example, the individual and their carer, may be appropriate (Care Act section 6(3)). In addition, in cases where the individual has significant difficulty in representing themselves at assessment and has no suitable advocate, the local authority is required to provide an independent advocate regardless of the individual's capacity under the Mental Capacity Act 2005.^5^

### Stage 2: assessing eligibility

At the eligibility stage, the Care Act replaces previous Fair Access to Care (FACS) guidance on eligibility criteria^6,7^ with a national eligibility threshold based on the causes, extent and impact of the individual's needs (~Box 3). It should however be noted that local authorities are able, where considered appropriate, to meet non-eligible needs and may choose to do so, for example, in order to prevent further deterioration.

#### Well-being, individual personal outcomes and eligibility

In order to judge eligibility, impacts and contexts of needs are relevant: individual needs must be considered against the specified outcomes to determine whether or not they can be met, and reasons for this should be understood. Some social needs are not addressed by the Care Act, for example housing and debt. A homeless person would not be eligible purely by virtue of their homelessness, but the reasons for homelessness may make them eligible. For example, becoming homeless solely through relationship breakdown would not be considered potentially eligible. However, homelessness through an inability to manage a tenancy due to the impact of severe mental illness (i.e. impaired ‘ability to maintain a habitable home’ as defined in the specified outcomes) may well be eligible.

Furthermore, the impact on well-being is a personalised assessment and an individual perception, so that two people with the same needs in relation to specific outcomes could end up with a different assessment of eligibility. For example, obsessive-compulsive disorder could manifest in repetitive behaviours which affect an individual's ability to maintain family relationships and employment. These two specified outcomes may be fundamental for one person and significantly affect their well-being. Another person may have very different priorities or personal outcomes that they want to achieve; their well-being is not significantly affected and they would not be eligible.

The word ‘significant’ is not defined in the legislation, rather it is a judgement made by the local authority after considering the person's needs and what is important to them. However, well-being is a broadly defined and holistically assessed concept, relating to areas such as: personal dignity; physical and mental health and emotional well-being; protection from abuse and neglect; control by the individual over day-to-day life; participation in work, education, training or recreation; social and economic well-being; domestic, family and personal relationships; suitability of living accommodation; and the individual's contribution to society.^5^

**Box 3** The National Eligibility Threshold (Regulation 2(1)14)^11^

An adult's needs meet the eligibility criteria if –

- the adult's needs arise from or are related to a physical or mental impairment or illness;
- as a result of the adult's needs the adult is unable to achieve two or more of the outcomes […] and
- as a consequence there is, or is likely to be, a significant impact on the adult's well-being.

The key to assessment under the Care Act is understanding what personal outcome (personal aim, wish or goal) the individual is looking for and what their needs are, before considering how these needs can be met. Person-centred assessment focuses on the individual and the difficulty they have in achieving personal outcomes, balanced with their strengths and support network; it does not start with service provision. For example, the assessment does not begin with ‘the person needs residential care’, but rather may find that ‘the person is unable to wash, dress and feed themselves. This is having an impact on their personal dignity and their ability to continue to live safely in their own home’.

The local authority must consider what strengths, resources and capabilities the person has themselves and within their support networks and wider community. This strengths-based approach to assessment and care planning can maximise opportunities for utilising assets found within communities and normative support networks, thereby reducing dependence on service provision by meeting people's needs in more innovative and creative ways.

### Stage 3: care planning

In developing and delivering preventive approaches to care and support, local authorities should ensure that individuals are not seen as passive recipients of support services, but are actively encouraged and supported to participate and are able to design care and support based around achievement of their goals. All assessments and subsequent care and support plans should be person-centred and genuinely engage the person and people involved in their care throughout.

Support plans should consider the broader needs identified by the assessment as well as the personal outcomes associated with the specified outcomes the individual is looking to achieve to maintain or improve their well-being. The person's own capabilities, assets and strengths and the potential for improving their skills, as well as the role of any support from family, friends or others that could help them to achieve what they wish for, should be incorporated into the plan. A person's independence should be maximised across these networks before any statutory service provision is considered to meet the desired personal outcomes.

Any person who requires ongoing support and has eligible needs is entitled to have these needs met through a personal budget. The Care Act 2014 has given parity to carers, who are now entitled to have their own eligible needs met through a carer's personal budget. A personal budget is an amount of money allocated for a person's support; this can be managed with or on behalf of the individual by the local authority or a broker, or can be paid to the individual as a direct payment. Person-centred care and support planning means that a person can receive part or all of their personal budget as a direct payment. Direct payments aim to enable a person to exercise the maximum possible choice over how they are supported, who they are supported by and where they are supported. The person must understand how much money is likely to be required to meet their eligible needs and have clear and realistic expectations of what is available locally. People who self-fund are entitled to receive necessary information, advice and support with support planning.

The third national TLAP survey^8^ demonstrated that over three-quarters of personal budget holders reported a positive impact of personal budgets on their lives. People with mental health difficulties were more likely than other groups to report a positive impact on relationships with carers, family and friends. However, older people were less likely than other cohorts to report a positive impact on mental health.

## Review

Plans may be revised as a scheduled review or in response to changing needs or circumstances. The review should be a positive opportunity to consider whether the plan is enabling the person to meet their needs and achieve their desired personal outcomes. At this point it can be considered whether the support provided is working (be this through a carer, the community, through a direct payment or a commissioned service through a personal budget), whether new personal outcomes need to be defined, or whether any changes need to be made to care and support to achieve improvement.

## Responsibilities of professionals

The Care Act places a responsibility on the local authority to inform the individual of their eligibility determination and produce a written record of whether any of their needs meet the eligibility criteria, and the reasons for this decision. Where an individual does not have eligible needs, the local authority must also provide information on what support may be available in the wider community, or what preventive measures might be taken to prevent or delay the condition progressing. This will require professionals responsible for eligibility decision-making to clearly evidence the reasons for their decisions and present these in an accessible format for the person concerned.

## Implications for service users and carers

The Care Act 2014 has changed the ability that a service user or carer has to influence assessment of their own needs and eligibility. Whereas the FACS criteria^7^ considered the needs of the individual, they did not consider their whole well-being and how this fits into their everyday lives, meaning that some service users may not have completely fitted into the specified categories. The criteria that the Care Act 2014 looks at focus on the individual in context, so that the impact on their well-being cannot now be overlooked or misjudged.

This holistic approach is mirrored by the TLAP ‘I’ statements, which make their markers for change much simpler to understand across a diverse range of individuals (Box 4). These statements complement the Care Act in allowing the individual service user to take control of everything that supports their specific needs and requirements. ‘I’ statements are presented in the form of first-person statements, for instance, ‘I have the information and support I need in order to remain as independent as possible’.

‘A service user or carer can automatically feel comfortable in all the statements as they are very clear and acknowledging. They allow you to take control of everything that supports your needs and requirements. The implications are quite dramatic; you feel worthwhile and not a burden to anyone and it allows you to take greater control of your own personal needs.’ (Deb Barnes)

**Box 4** Think Local, Act Personal ‘I’ statements^9^

- Information and advice: having the information I need, when I need it
- active and supportive communities: keeping friends, family and place
- flexible integrated care and support: my support my own way
- workforce: my support staff
- risk enablement: feeling in control and safe
- personal budgets and self-funding: my money.

## Dilemmas and challenges

The Care Act 2014 has introduced some major statutory changes to the way social care is delivered nationally, and as such presents a number of dilemmas and challenges to service users, carers and service providers.

### Assessment as intervention

Assessment under the Care Act should be an intervention in itself rather than merely a process by which individuals are granted or denied access to funded services. This presents challenges both for local authorities as a whole and for individual professionals in a number of areas, including the necessary provision of reliable and up-to-date information about local services, and management of the time and resources required to ensure that assessments can be completed in a full and holistic manner.

### Provision of appropriate and proportionate assessment

Assessment under the Care Act 2014 requires local authorities to become more flexible in administering assessments and to develop assessment processes which allow for this both internally and in collaboration with other organisations.

### Measurement of efficacy

Whereas the Care Act 2014 defines specified outcomes for service users and carers, the way in which these are met will be highly specific to each individual service user and may create challenges in the ways local authorities monitor and measure the efficacy of service delivery.

### Provision of services

Local authorities are expected under the Care Act to promote and shape the local market so as to achieve diverse provision of care and support in their area. This carries with it budgetary implications with regards to commissioning, funding and fee-setting, which must be considered not only in terms of local authority budgets but also in relation to providers' sustainability.^10^

### Carer support

The Care Act broadens previous definitions of the carer role and requires assessment of support needs for anyone who feels that they fulfil this role.^6^ The challenge for local authorities is to provide sufficient information to all potential carers on their rights to assessment and possible financial support, while managing the potentially increased demand for these assessments and provisions.

### Conclusion

Time will tell whether the aspirations of the Care Act are achieved. The emphasis is clear that care should be holistic and empowering; promotion of well-being is at its core. It is hoped that it will develop services that are inclusive, work in a person-centred way, and achieve specific outcomes. The introduction of the Care Act makes this way of working a statutory duty that public services will be measured by. It will be through individual lives and stories that success will be realised.

## References

[1] Lamb N Care Bill becomes Care Act 2014. Department of Health, 2014.

[2] Department of Health No Secrets: Guidance on Developing and Implementing Multi-Agency Policies and Procedures to Protect Vulnerable Adults from Abuse. Department of Health, 2000.

[3] Robertson R Gregory S Jabbal J The Social Care and Health Systems of Nine Countries. The King's Fund, 2014.

[4] Schwer B How the Care Act 2014 will shape assessment and support planning – a legal opinion. Community Care 2014, 27 2.

[5] Department of Health Care and Support Statutory Guidance Act. Issued under the Care Act 2014. 6 2014 (updated October 2016) https://www.gov.uk/government/publications/care-act-statutory-guidance/care-and-support-statutory-guidance

[6] Department of Health Prioritising Need in the Context of Putting People First: A Whole System Approach to Eligibility for Social Care. Guidance on Eligibility Criteria for Adult Social Care, England 2010. Department of Health, 2010.

[7] Social Care Institute for Excellence Fair Access to Care Services (FACS): Prioritising Eligibility for Care and Support. SCIE, 2013

[8] Waters J Hatton C Third National Personal Budget Survey: Experiences of Personal Budget Holders and Carers across Adult Social Care and Health. In Control, Lancaster University & TLAP, 2014.

[9] Think Local Act Personal Making it Real: Marking Progress towards Personalised, Community Based Support. TLAP, 2011 (http://www.thinklocalactpersonal.org.uk/_assets/NEWMakingItReal.pdf).

[10] Local Government Association Guide to the Care Act 2014 and the Implications for Providers. Local Government Association, 2015.

[11] Care and Support (Eligibility Criteria) Regulations 2014 http://www.legislation.gov.uk/ukpga/2014/23/section/13/enacted

